# Supplementary material for: Mediterranean Diet and Lung Function in Adults Current Smokers: A Cross-Sectional Analysis in the MEDISTAR Project
Source: Nutrients. 2023 Mar 3;15(5):1272. doi: 10.3390/nu15051272 (PMC10005310; doi:10.3390/nu15051272)
Supplement: Supplementary file 1 [file nutrients-15-01272-s001.zip › nutrients-2228485-supplementary.pdf]

Supplementary Materials

Table S1 shows the positive answers given by the participants and the influence of each question on the total score of the MEDAS questionnaire, according to the degree of adherence to the MeDi. As can be seen, the only question that has not obtained statistical significance and that there are no differences between the three adherence groups is the 5th question, referring to red meat. The low adherence group is significantly different from the medium and high adherence group in questions 1, 7, 10, 13 (on olive oil consumption as main culinary fat, soda drinks, fish or seafood and poultry more than red meat). The high adherence group is significantly different from the low and medium adherence group in questions 2, 3, 4 (consumption of olive oil more than 4 tablespoons/day, vegetables and fruits). In the rest of the questions, all the adherence groups were different from each other.

Table S1. Participants with positive answer (%) to each of the 14 Items of MEDAS, according to Mediterranean Diet Adherence group.

	**Low Adherence**	**Medium Adherence**	**High Adherence**	***p* Value**	**Post-Hoc Test**
Questionnaire	(*n* = 96)	(*n* = 161)	(*n* = 146)		
Q 1, use olive oil as main culinary fat	86 (89.6%)	157 (97.5%)	144 (98.6%)	0.002	a,b
Q 2, olive oil >4 tablespoons	55 (57.3%)	102 (63.4%)	127 (87.0%)	<0.001	b,c
Q 3, vegetables ≥ 2 servings/day	14 (14.6%)	37 (23.0%)	73 (50.0%)	<0.001	b,c
Q 4, fruits ≥ 3 servings/day	5 (5.2%)	17 (10.6%)	45 (30.8%)	<0.001	b,c
Q 5, red or processed meats < 1/day	48 (50.0%)	88 (54.7%)	92 (63.0%)	0.111	
Q 6, butter/cream/margarine <1/day	57 (59.4%)	133 (82.6%)	138 (94.5%)	<0.001	a,b,c
Q 7, soda drinks < 1/day	50 (52.1%)	139 (86.3%)	127 (87.0%)	<0.001	a,b
Q 8, wine glasses ≥ 7/week	7 (7.3%)	56 (34.8%)	68 (46.6%)	<0.001	a,b,c
Q 9, legumes ≥ 3/week	10 (10.4%)	23 (14.3%)	75 (51.4%)	<0.001	b,c
Q 10, fish or seafood ≥ 3/week	10 (10.4%)	55 (34.2%)	64 (43.8%)	<0.001	a,b
Q 11, commercial bakery ≤ 2/week	32 (33.3%)	103 (64.0%)	125 (85.6%)	<0.001	a,b,c
Q 12, nuts ≥ 3/week,	11 (11.5%)	50 (31.1%)	69 (47.4%)	<0.001	a,b,c
Q 13, poultry more than red meats	50 (52.1%)	141 (87.6%)	135 (92.5%)	<0.001	a,b
Q 14, use of *sofrito* sauce ≥ 2/week	63 (65.6%)	128 (79.5%)	130 (89.1%)	<0.001	a,b,c

(*) A bivariate analysis was made using the Chi-square test and a post-hoc test (Benjamini and Hochberg) to compare the differences between the 3 adherence groups and the 2 to 2 differences between the groups: (a) p-value < 0.05 when comparing low against medium adherence; (b) p-value < 0.05 when comparing low to high adherence; (c) p-value < 0.05 when comparing medium with high adherence. MEDAS refers to the Mediterranean Diet Adherence Score validated by the PREDIMED study. Adherence to the Mediterranean Diet was assessed using the 14-item questionnaire that considers low (0–6 points), medium (7–8 points) and high (9–14 points) adherence [19].
